# Supplementary material for: Meta-analysis of the efficacy of the erector spinae plane block after spinal fusion surgery
Source: PeerJ. 2024 Oct 30;12:e18332. doi: 10.7717/peerj.18332 (PMC11531255; doi:10.7717/peerj.18332)
Supplement: Supplemental Information 2 [file peerj-12-18332-s002.docx]

Table S1 Search strategy

| Search number | Query |
| --- | --- |
| 1 | "Spinal Fusion"[Mesh] |
| 2 | (((((((("Spinal Fusion"[Mesh]) OR (Spinal Fusion[Title/Abstract])) OR (Fusion, Spinal[Title/Abstract])) OR (Fusions, Spinal[Title/Abstract])) OR (Spinal Fusions[Title/Abstract])) OR (Spondylodesis[Title/Abstract])) OR (Spondylodeses[Title/Abstract])) OR (Spondylosyndesis[Title/Abstract])) OR (Spondylosyndeses[Title/Abstract]) |
| 3 | ((Erectorspinae[Title/Abstract]) OR (ESP[Title/Abstract])) OR (ESP block[Title/Abstract]) |
| 4 | ((((((((("Spinal Fusion"[Mesh]) OR (Spinal Fusion[Title/Abstract])) OR (Fusion, Spinal[Title/Abstract])) OR (Fusions, Spinal[Title/Abstract])) OR (Spinal Fusions[Title/Abstract])) OR (Spondylodesis[Title/Abstract])) OR (Spondylodeses[Title/Abstract])) OR (Spondylosyndesis[Title/Abstract])) OR (Spondylosyndeses[Title/Abstract])) AND (((Erectorspinae[Title/Abstract]) OR (ESP[Title/Abstract])) OR (ESP block[Title/Abstract])) |

Table S2 GRADE

**Question:** Erector spinae plane block compared to control for spinal fusion surgery


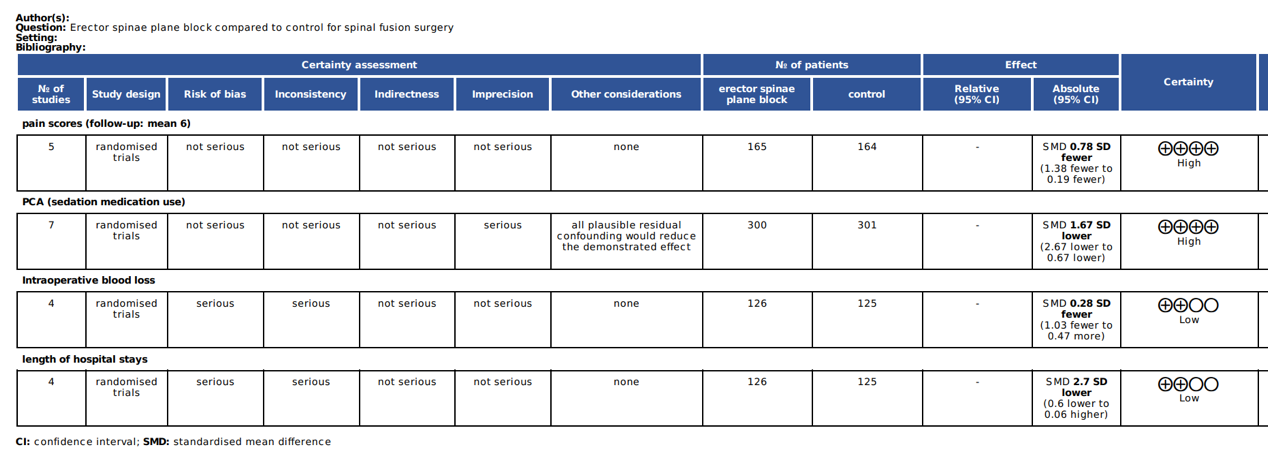


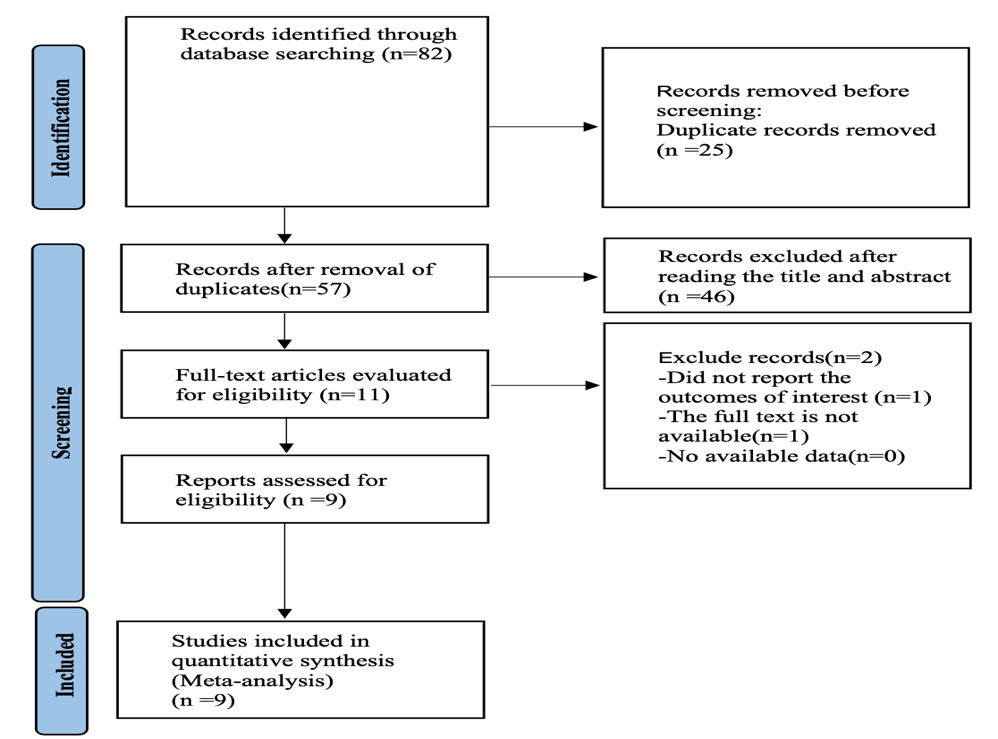


Figure S1 Literature search flow chart


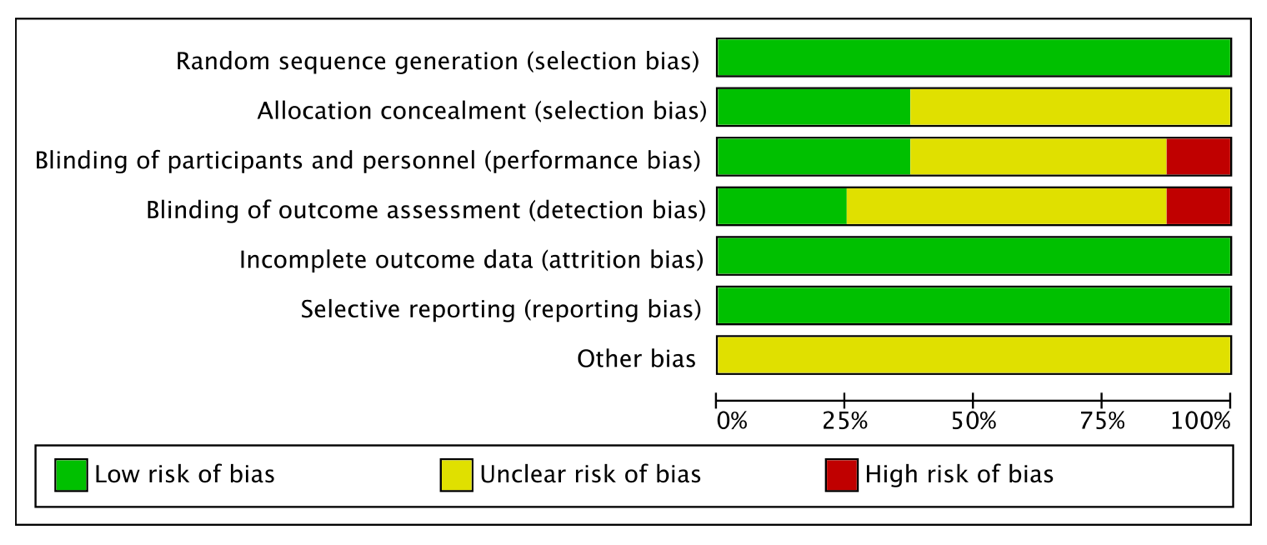


Figure S2 Risk of bias graph
