# Supplementary material for: Meta-analysis of the efficacy of the erector spinae plane block after spinal fusion surgery
Source: PeerJ. 2024 Oct 30;12:e18332. doi: 10.7717/peerj.18332 (PMC11531255; doi:10.7717/peerj.18332)
Supplement: Supplemental Information 3 [file peerj-12-18332-s003.docx]

Why did you do this study?

this is the first time that meta-analysis was used to evaluate the effect of EPSB on spinal fusion surgery, and our study found that EPSB was able to reduce postoperative pain after spinal fusion surgery and was able to reduce the use of sedative medication, but it had no effect on intraoperative blood loss or length of hospital stay.

What does this study add to other studies?

Meta-analysis showed that EPSB could reduce pain scores at 2h [SMD=-0.78, 95% CI (-1.38, -0.19), GRADE: Moderate], 6h [SMD=-0.81, 95% CI (-1.23, -0.38), GRADE: Moderate], 12h[SMD=-0.59, 95% CI (-1.05, -0.13), GRADE: Moderate], 24h [SMD=-0.54, 95% CI (-0.86, -0.21), GRADE: Moderate], 48h[SMD=-0.40, 95% CI (-0.75, -0.05), GRADE: Moderate] after spinal fusion surgery, as well as the PCA (sedation medication use) [SMD=-1.67, 95% CI (-2.67, -0.67), GRADE: Moderate]. However, EPSB had no effect on intraoperative blood loss [SMD=-0.28, 95% CI (-1.03, 0.47), GRADE: Low] and length of hospital stay [SMD=-0.27, 95% CI (-0.60, 0.06), GRADE: Low].
